# Is the Scale for Measuring Motivational Interviewing Skills a valid and reliable instrument for measuring the primary care professionals motivational skills?: EVEM study protocol

**DOI:** 10.1186/1471-2296-13-112

**Published:** 2012-11-22

**Authors:** Luis Á Pérula, Manuel Campiñez, Josep M Bosch, Nieves Barragán Brun, Juan C Arboniés, Julia Bóveda Fontán, Remedios Martín Alvarez, Jose A Prados, Enrique Martín-Rioboó, Josep Massons, Margarita Criado, José Á Fernández, Juan M Parras, Roger Ruiz-Moral, Jesús Manuel Novo

**Affiliations:** 1 Unidad Docente de Medicina Familiar y Comunitaria de Córdoba, Instituto Maimónides de Investigación Biomédica de Córdoba (IMIBIC)/Hospital Universitario Reina Sofía/Universidad de Córdoba, Avda. Menéndez Pidal, s/n, Córdoba, Spain; 2 CAP Vallcarca, Barcelona, Spain; 3 Área Básica de Salud Encants Maragall, Institut Catala de la Salut (ICS), Barcelona, Spain; 4 EAP Colmeiro, C/ Pintor Colmeiro, 11, Vigo, Spain; 5 Centro de Salud de Beraun- Errenteria, Avda. Galzaraborda 67, Donostia, Spain; 6 Centro de Salud Lucano (Córdoba), Servicio Andaluz de Salud, Spain; 7 ABS Mataró 7. Institut Catala de la Salut (ICS), Barcelona, Spain; 8 Centro de Saúde "A Milagrosa", Lugo, Spain

**Keywords:** Motivational interviewing, Behavior change counseling, Lifestyle change), Dyslipidemia, Cardiovascular risk factors

## Abstract

**Background:**

Lifestyle is one of the main determinants of people’s health. It is essential to find the most effective prevention strategies to be used to encourage behavioral changes in their patients. Many theories are available that explain change or adherence to specific health behaviors in subjects. In this sense the named Motivational Interviewing has increasingly gained relevance. Few well-validated instruments are available for measuring doctors’ communication skills, and more specifically the Motivational Interviewing.

**Methods/Design:**

The hypothesis of this study is that the Scale for Measuring Motivational Interviewing Skills (EVEM questionnaire) is a valid and reliable instrument for measuring the primary care professionals skills to get behavior change in patients. To test the hypothesis we have designed a prospective, observational, multi-center study to validate a measuring instrument. –Scope: Thirty-two primary care centers in Spain. -Sampling and Size: a) face and consensual validity: A group composed of 15 experts in Motivational Interviewing. b) Assessment of the psychometric properties of the scale; 50 physician- patient encounters will be videoed; a total of 162 interviews will be conducted with six standardized patients, and another 200 interviews will be conducted with 50 real patients (n=362). Four physicians will be specially trained to assess 30 interviews randomly selected to test the scale reproducibility. -Measurements for to test the hypothesis: a) Face validity: development of a draft questionnaire based on a theoretical model, by using Delphi-type methodology with experts. b) Scale psychometric properties: intraobservers will evaluate video recorded interviews: content-scalability validity (Exploratory Factor Analysis), internal consistency (Cronbach alpha), intra-/inter-observer reliability (Kappa index, intraclass correlation coefficient, Bland & Altman methodology), generalizability, construct validity and sensitivity to change (Pearson product–moment correlation coefficient).

**Discussion:**

The verification of the hypothesis that EVEM is a valid and reliable tool for assessing motivational interviewing would be a major breakthrough in the current theoretical and practical knowledge, as it could be used to assess if the providers put into practice a patient centered communication style and can be used both for training or researching purposes.

**Trials registration Dislip-EM study:**

NCT01282190 (ClinicalTrials.gov)

## Background

Lifestyle is one of the main determinants of people’s health. It is essential to find the most effective prevention and health-promotion strategies to be used by general practitioners to encourage behavioral changes in their patients.

Many theories are available that explain change or adherence to specific health behaviors in subjects. Many of these theories have resulted in actions that include brief advice and counseling of different intensity and frequency. In this sense, recently, the named “Motivational Interviewing” (MI) has increasingly gained relevance and, in some clinical situations, efficiency [1]. MI was developed as a way to help people work their ambivalence and provoke change [2]. It was a transtheoretical model derived from the Person-Centered Therapy, which combined an empathetic and understanding style of counseling [3] with a straightforward method for resolving ambivalence in the direction of changing. Theoreticians have made this theory evolve by deepening their study of MI, which is currently defined as "a style of clinical skills for understanding patients’ motivations for behavior change in the interest of their health" [4].

Finding solid evidence of MI effectiveness –which is currently being assessed by our research group-, is as important as having instruments available to assess to what extent clinicians use the motivational approach when interviewing their patients, and whether they use it properly or not. To detect the skills of the clinicians involved in clinical trials carried out for assessing the effectiveness of MI, is an important task. In this sense, the contributions published so far are very limited in number. There are two relevant instruments for assessing the abilities/techniques of the most orthodox and extended theoretical approach: that of Miller and Rollnick’s [4], as concerns MI, the Motivational Interviewing Skill Code (MISC) [5] and its abridged and enhanced version, the Motivational Interviewing Treatment Integrity (MITI) [6]; and the Behavior Change Counseling Index (BECCI) [7]. The former has only recently been proved to be reliable and effective in assessing MI skills and could be used both for clinical and research purposes [8,9]. Scant studies have been published on the implementation of these instruments in Spain. There is only a research paper published by Spanish-speaking experts, although it has some methodological limitations that make it hardly adaptable [10].

This is the reason why new instruments should be developed and tested, with the purpose of measuring general practitioners’ motivational skills when it comes to encourage behavior change in patients. Such instruments should be based on three aspects: 1) An eclectic approach including different strategies and skills suggested in different theories, which would allow researchers to assess the effectiveness of each of such instruments. This approach can be more useful for clinicians than other tools, which might be theoretically too rigid. 2) The instrument should be adapted to our social and cultural environment, and it should be proved effective in general practice, where these approaches, as well as their limitations and possibilities, are more frequently used and well known. For example, due to the primary care burden, professionals have difficulty in exceeding the usual average time per interview (which in Spain is about seven minutes). [11] Therefore, it would be unrealistic to propose interventions of about one hour long, as those suggested by MITI. 3) Finally, in this context, any translation and adaptation of the existing tools would not represent a significant step forward and would involve a similar effort to that required for developing a new measurement scale.

In Spain, our research group has recently developed two scales (GATHA-Res, CICAA) [12,13] for identifying clinical interviewing skills to improve clinical practice in trainers and researchers, which are based on a generic approach to primary care known as "patient-centredness" [14]. The aim of this new approach is to focus on the ideas, beliefs and expectations that patients have regarding their condition, and on the possible action plans to be undertaken, while promoting patients’ involvement in decision-making. These scales have rendered good psychometric indexes, and have proved useful for the purposes intended. At the same time that they have also served as a basis for other more specific purposes, such as patient involvement in decision-making (CICAA-D) [15].

For all these reasons, it would seem reasonable and relevant to use "patient-centered" clinical interaction as a starting point. This approach focuses on the relevance of building a “therapeutic empathy” with the patient -which meets MI principles. Similarly, it is also reasonable to found this new instrument on the same methodological principles as previous instruments, which were based on the assumption that clinical communication is a set of conducts that can be observed and measured as any other clinical skill [16]. Therefore, such conducts should meet a number of requirements that are well-known in the context of primary care communication [17]. Thus, a good measurement tool should be based on a well-defined model of professional-patient relationship, as well as include multiple observable categories (multidimensionality), and have appropriate and well-documented psychometric properties (validity and reliability), as well as practicability. Further, it is noteworthy that one of the most reliable assessment methods is used in this instrument: the evaluation of recorded interviews by external trained raters [18].

With this project we try to test the hypothesis that a tool called "Assessment Scale motivational interviewing" (EVEM in Spanish) designed to assess whether the Spanish doctors have MI skills to promote in their patients behavioural changes have good psychometric properties, in terms of validity and reliability.

## Methods

The conceptual hypothesis has formulated so: “Is the Scale for Measuring Motivational Interviewing Skills (EVEM, acronym in Spanish of “Escala de Valoración de la Entrevista Motivacional”) a valid and reliable instrument to measure the skills of primary care professionals ?”.

The main objective of this study, therefore, is to validate the EVEM questionnaire for its use in clinical interviewing with patients suffering from dyslipidemia.

The specific objectives are:

1. Developing a scale and assess its qualitative validity;

1.a) Developing and identifying scale items -basing on the theoretical model- and preparing a draft questionnaire for assessing general practitioners’ motivational interviewing skills;

1.b) Conducting an assessment based on expert opinions: face, consensual and content validity;

2. Analyzing the psychometric properties and attributes of the scale:

2.a) Assessing the questionnaire’s validity and reliability:

2.a.1.-in terms of reproducibility and variability (inter-observer reliability, intra-observer agreement -test-retest);

2.a.2.-in terms of homogeneity (scale internal consistency);

2.b) Assessing the generalizability of EVEM reliability;

2.c) Assessing its dimensionality (factor analysis);

2.d) Refining the scale contents (response rate, item-total correlation, reliability).

2.e) Assessing the scale’s sensitivity to change.

### Study design

**Figure 1:**
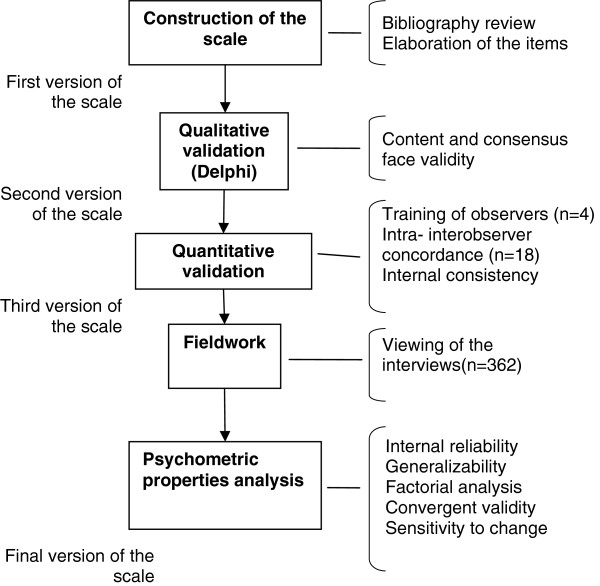
Scheme of the EVEM study design.

For testing our hypothesis we used two epidemiological designs: A qualitative study in its first stage (expert opinions, Delphi technique), and a quantitative, prospective, observational and multicenter study in its second stage. Figure 1 shows the study protocol design. This study approaches the complementary objective of the Dislip-EM project, a cluster randomized, controlled clinical trial designed to assess the effectiveness of Motivational Interviewing in reducing cardiovascular risk and improving lipid control in patients with hyperlipidemia [19].

### Action plan

The procedure in each stage is as follows:

-Stage 1: design of a questionnaire (Objective 1.a). The scale is designed in consistency with the underlying theoretical model, with the analysis of the scientific literature available, and with scientific evidence on clinical communication [3,5,6,20].

-Stage 2: qualitative validation (objective 1.b). To analyze the face and consensual validity of the questionnaire, a group of 15 key-informants specially trained in clinical communication was created. Then, Delphi-type methodologies were implemented in an iterative process of three e-mail rounds [21]. Once this stage was completed, three versions were obtained from the first EVEM version (see Additional file 1). Further, a manual was prepared for the evaluator in two formats: an extended version and an abridged version.

-Stage 3: psychometric properties and attributes of the scale (Objective 2): Objective 2.a): intra-/inter-observer agreement/reliability:

2.a.1. Intraobserver reliability: four evaluators (two experts and another two non-experts in MI -previously trained in the use of EVEM- will assess twice, separately and independently the same sample of 18 interviews randomly selected –from the 162 interviews done with standardized patients- in a 4–6 week interval. Evaluators are general practitioners with clinical experience and advanced theoretical/practical knowledge on MI.

2.a.2. Interobserver reliability: for training purposes, expert raters will invite non-expert to watch motivational interviewing videos of different models (stages of change) and quality, for providing feedback to the EVEM scale. Both non-expert observers and the two experts will independently assess the 18 interviews to assess interobserver agreement.

2.b) Concomitantly, the reliability-generalizability coefficients will be estimated. Considering the results obtained by our research group in the validation process of a questionnaire for assessing patient-centered interviews, such coefficients are expected to be between six and nine interviews [13].

## Statistical analysis

### Stage 2: qualitative analysis (objective 1.b)

In the third Delphi round, the relevance/value of the questionnaire items was weighted by using a five-degree ordinal score scale. From the results obtained, the arithmetic mean, mode, distribution intervals and variance of each item were estimated as a way to measure the level of agreement. Therefore, high variance would represent a low level of agreement on the relevance of each item, and vice versa.

### Stage 3: psychometric properties and attributes of the scale (objective 2)

To assess intra-/inter-observer agreement levels, the simple concordance index (SCI), the global concordance index and Cohen’s kappa index (κ) will be estimated [22]. Absolute values will be provided, along with confidence intervals and p value (p < 0.05). Intraclass correlation coefficients will also be estimated [22,23]. The results obtained will serve to assess the level of agreement for each task and dimension, and for the total score of the EVEM questionnaire.

Further, the Student’s-*t*-test will be performed to compare the pairs of means obtained from the total scale scores, and the Bland & Altman methodology will be implemented. The latter is a descriptive method for analyzing graphically the pattern of deviations from zero (total agreement) and for determining whether intraobserver differences decrease or not as the average score increases [24]. To assess any potential relative bias (systemic difference between both observations), the means of the differences between the values obtained in each interview, their standard deviations (SD) and limits of agreement (mean of the differences ± 1.96 x SD) will be estimated.

To express reproducibility by the κ statistic, the classification suggested by Landis & Koch will be applied [25]. Landis & Koch characterized κ <0.40 values as indicating slight agreement, 0.75-0.40 as good, and κ>0.75 as excellent agreement. For the interpretation of the intraclass correlation coefficient (ICC), the classification established by Jiménez will be used [26], which characterizes ICC >0.90 as very good reproducibility; 0.71-0.90 as good; 0.51-0.70 as moderate; 0.31-0.50 as slight and <0.31 as poor or no reproducibility.

To assess the questionnaire’s homogeneity or internal agreement, Cronbach’s alpha coefficient (α) will be used. The extent to which the α coefficient changes when an item is removed will also be analyzed, to determine whether the questionnaire’s reliability is altered or not, and to evaluate whether a specific item should be discarded [22,27].

The dimensionality of the EVEM scale will be analyzed by factor analysis, which will provide statistics on the underlying scale factors of the theoretical construct to be examined [28]. Since there are no prior hypotheses on the specific components of the construct or on their potential correlation, an exploratory –rather than a confirmatory- analysis will be performed. The stages proposed by Norman & Streiner [29] will be followed for factor analysis. The correlation coefficient between the different variables will be estimated by the Kaiser-Meyer-Olkin measure of sampling adequacy (KMO) and by Barlett’s test of sphericity.

The generalizability of the EVEM scale will be measured in each observer by the ICC, and the reliability of the scale total score will be assessed in a consecutive case series study, following Mercer et al. method [30]. In competence measurement studies, for an evaluator to be considered reliable, it is agreed that they must obtain ICC values >0.856 [31].

Subsequently, the scale content will be refined. The response rate and item-total correlation will be estimated to test the discrimination capacity of the items. Finally, the item-total correlation will be computed by identifying the correlation between Pearson’s coefficient value for each individual item and the total score for the questionnaire, task and dimension or factor where the item was included, once the item has been discarded [22]. It was determined that items with coefficients <0.30 would be discarded [32].

The construct validity will be analyzed and the discrimination capacity of the scale will be determined by using the extreme-groups methodology [33]. On such purpose, the two randomized groups created for the Dislip-EM project will be used: in one of the groups, clinicians will perform MI sessions (n=110), and in the other group, they will perform common clinical interviewing with their patients (no-MI group) (n=110). The correlation between the scale and other variables will also be assessed. Finally, the correlation between the scores obtained with the EVEM scale and the endpoints obtained with the “parent” study (Dislip-EM project) will be analyzed.

Sensitivity to change will be assessed by two methods:

a) By measuring the impact of special training: the hypothesis will test whether the GPs in the Intervention Group (IG) improve their MI skills after joining the training program. Significant statistical differences are expected to be found between the mean scores obtained on the EVEM scale by the Intervention Group and those obtained by the professionals in the Control Group (CG).

Further differences are also expected in the Experimental Group (EG) subjects before and after participating in the training program. On such purpose, standard interviews performed by the IG with standardized patients will be recorded before and after joining the training program; similarly, the same type of interview will be performed –and recorded- by the CG before undertaking fieldwork (162 interviews in total). All the interviews will be assessed by a trained evaluator.

b) Impact on patients’ health 12 months after the first intervention. The composite endpoint will be Cardiovascular Risk, measured with SCORE and REGICOR tables, which are both validated for the Spanish population [34-36]; similarly, cholesterol levels –which are the primary endpoint of this study- will also be analyzed. In both cases, Pearson’s correlation test will be performed (p ≤ 0.05).

### Participants

A total of 50 GPs from 50 offices at 32 primary care centers of the Spanish National Health System are participating in the Dislip-EM study [19]. The following subpopulations are being studied:

-Subpopulation 1: To test the expert-expert, evaluator-evaluator intraobserver reliability, and the expert-evaluator interobserver reliability, a sample of 18 interviews with standardized patients will be performed (eight of which will be EG patients -where GPs are expected to perform MI-, and another eight with CG patients -where GPs are not expected to perform MI). The purpose of using two subsamples is to ensure the collection of different levels of performance concerning the model that the instrument must record and discriminate. The interviews will be selected at random from the 162 that have already been performed with simulated patients.

-Subpopulation 2: For the purposes of this study, each GP will recruit eight-nine regular patients. Each clinician is expected to video record their encounters with a patient selected randomly, in their visits at 2, 4, 8 and 12 months so, at the completion of this stage, 200 interviews will be available in video format. The analysis of the different types of validity (content, construct, sensitivity to change) will be performed by using the samples from both subpopulations (n=162 from the simulated interviews plus 200 from real interviews, making a total of 362 interviews). The assessment of such interviews by the EVEM tool will be conducted by one of the expert physicians.

### Sample size

The sampling unit is the video recording of a clinical interview. From a general approach, most of authors agree that, in questionnaire validation studies, a sample size of 200–300 or 5–10 participants (in this case, clinical interviews) per scale variable would be adequate [22,27,29]. Thus, considering that the last version of the scale has 16 items, the number of interviews required would be 80–160.

To calculate the sample size required to estimate inter-/intra-observer agreement, the formula developed by Norman & Streiner [30] was used. They establish that the desirable minimum sample size can be estimated by the formula n= 2 x C^2^, where C is the number of answer categories. As the scale has three answer categories, the desirable minimum sample size is 18 interviews.

### Protection against bias

Logically, the clinicians involved in the study might feel observed, which could affect their performance and modify their behavior. However, but there is ample experience in the use of observation methods similar to the one used in this study confirming that, in real practice, it is very difficult that a clinician modifies his/her interviewing style. Variations in patients are still more unusual [37]. Anyway, in the event that bias would occur, it would not essentially affect the purpose of this study, as its objective is to test the psychometric properties of the scale, regardless of whether these are more or less consistent with what would happen if they were conditioned by the observation method.

## Discussion

To prove the hypothesis that EVEM has good psychometric properties. If so, this will allow to apply this questionnaire for assessing the results derived from any research study with the aim of assess possible behavioral changes in health habits as a result of a motivational approaching. In Clinical practice we would have an specific instrument for these purposes. The motivational interviewing is being used increasingly in many important public health problems such as the prevention of cardiovascular risk factors (dislipaedemia, overweight and obesity, diabetes, high blood pressure,…), toxic habits (smoking, alcohol and illegal drug use), therapeutic adherence (one of the main reasons for treatment failure in chronic patients), sexual behaviours (prevention of sexually transmitted diseases, especially HIV), or mental health problems, so the tool would have a wide range of research applications.

### Ethical

This project has been approved by the Commission on Ethics and Health Research Center of Reina Sofia Hospital (Córdoba, Spain), dated 08/11/2010.

## Abbreviations

MI: Motivational Interviewing.; EVEM: Scale for Measuring Motivational Interviewing Skills (acronym in Spanish of “Escala de Valoración de la Entrevista Motivacional”).; GPs: General Practitioners; IG: Intervention Group.; CG: Control Group.; K: Cohen’s kappa index.

## Competing interests

The authors declare that they have no competing interests.

## Authors’ contributions

LAPT is the principal Investigator who conceived the study and led the study design and funding application. Contributed to the Statistical Analysis Plan. Led the writing of this manuscript. JMB, JBF, NBB, MCN, JCA, JAP, JM, RM, JAF, RR, JMP and JN contributed to the study design, funding application and study implementation. Contributed to writing the paper. MCN, JBF, JN and NBB will conduct the assessment process of the interviews for the analysis of inter-/intra- observer agreement. MCN will assess all the records to perform the validity analysis. All authors contributed to, read and approved the final version of the manuscript.

## Participants in the Collaborative Group of the *Dislip-EM Study*

1. Emilio García Criado (CS Fuensanta. Córdoba. Spain)

2. Enrique Martín Rioboó (CS Fuensanta.Córdoba)

3. María Pineda Alonso (CS Levante sur. Córdoba)

4. Ana Roldán Villalobos (CS Huerta de la Reina. Córdoba)

5. Antonio Pérez Fuentes (Consultorio Villafranca de Córdoba)

6. Mª José Acosta García (CS Adamuz. Córdoba)

7. Isabel de Andrés Cara (CS Levante sur. Córdoba)

8. Antonio León Dugo (CS Levante sur. Córdoba)

9. Pilar Serrano Varo (CS Posadas. Córdoba)

10. Antonio Valero Martín (Consultorio Villafranca de Córdoba)

11. Juan Manuel Parras Rejano (CS Peñarroya. Córdoba)

12. Rosana Izquierdo Fernández (CS Coruxo. Vigo)

13. Antonio Fernández Crespo (CS Pintor Colmeiro. Vigo)

14. Mª Dolores Pazo Ferreiro (CS Pintor Colmeiro. Vigo)

15. Susana Hernaiz Valero (CS Val Miñor. Vigo)

16. Mª Jesús Cobas Martínez (CS Matamá. Vigo)

17. Neus Fernández Danés (ABS Centre L’ Hospitalet de Llobregat. Barcelona)

18. Francisca Pérez Fuentes (CS Virgen Linarejos. Linares. Jaén)

19. Clara Soria López (CS Virgen Concha. Zamora)

20. Silvia Membrilla Pastor (CAP Ramona Vía.El Prat de Llobregat. Bareclona)

21. Francisco Mora Moreno (CS Molino de la Vega. Huelva)

22. José Luís Montero Monterroso (CS Fernán Núñez. Córdoba)

23. Mª Dolores Vargas Rubio (CS Fernán Núñez. Córdoba)

24. Antonio López Hernández (CS Posadas. Córdoba)

25. Santiago Avilés Cigüela (ABS Centre L’ Hospitalet de Llobregat. Barcelona)

26. Susana Aldecoa Landesa (Centro Saúde Beiramar. Barcelona)

27. Félix Suárez González (CS San Roque. Badajoz)

28. Cristina Aguado Taberné (CS Santa Rosa. Córdoba)

29. Manuel Rico Cabrera (CS Villaviciosa de Córdoba)

30. Francisco Caro Tejero (CS Bujalance. Córdoba)

31. Silvia Díez Moreno (CS Tui. Pontevedra)

32. Gina Ballester Adell (CAP Vallcarca Sant Gervasi. Barcelona)

33. Alexis Tena Domingo (CAP Vallcarca Sant Gervasi. Barcelona)

34. Juantxo Mendive Arbeloa (CAP La Mina.S. Adriá de Besos. Barcelona)

35. Azucena Carranzo Tomás (CAP Vallcarca Sant Gervasi.Barcelona)

36. Laura Belmonte Calderón (CS “El Castell”.Castelldefels. Barcelona)

37. Miriam Ruíz Sánchez (ABS Centre L’ Hospitalet de Llobregat. Barcelona)

38. Cristina Ortodó Parra (ABS Centre L’ Hospitalet de Llobregat. Barcelona)

39. Sonia Cibrián Sánchez (CAP Vallcarca Sant Gervasi. Barcelona)

## Pre-publication history

The pre-publication history for this paper can be accessed here:

http://www.biomedcentral.com/1471-2296/13/112/prepub

## Supplementary Material

Additional file 1 **Annex.** Evem 1.3 English version. Click here for file
