# Supplementary material for: Is the Scale for Measuring Motivational Interviewing Skills a valid and reliable instrument for measuring the primary care professionals motivational skills?: EVEM study protocol
Source: BMC Fam Pract. 2012 Nov 22;13:112. doi: 10.1186/1471-2296-13-112 (PMC3528408; doi:10.1186/1471-2296-13-112)
Supplement: Additional file 1 — Annex. Evem 1.3 English version. [file 1471-2296-13-112-S1.doc]

**Annex**

**Evem 1.3 English version**

| Id code:  Target behavior:  Total duration of the interview (min): |  |  |  |  |
| --- | --- | --- | --- | --- |
| In which way, the professional... | 0 | 1 | 2 | NA |
| 1. Establishes a good rapport with the patient through non-verbal communication |  |  |  |  |
| 2. Shows empathy at the appropriate moments |  |  |  |  |
| 3. Makes an adequate positioning of the patient in relation to the target behavior and evaluates his/her ambivalence |  |  |  |  |
| 4. Works according to the positioning of the patient through the interview |  |  |  |  |
| 5. Uses open questions |  |  |  |  |
| 6. Affirms the patient (abilities, aptitudes, effort, interest…) |  |  |  |  |
| 7. Uses reflective listening |  |  |  |  |
| 8. Makes summaries of the information given by the patient |  |  |  |  |
| 9. Facilitates change talk |  |  |  |  |
| 10. Has explored the degree of commitment from patient in relation to a possible behavior change |  |  |  |  |
| 11. Detects resistance to change and uses specific strategies to avoid and manage underlying resistance |  |  |  |  |
| 12. Offers information according to the patient’s problems and needs |  |  |  |  |
| 13. Promotes the delimitation and prioritization of the change objectives with the patient |  |  |  |  |
| 14. Negotiates a feasible action plan which considers the patients options |  |  |  |  |
| 15. Once the change has taken place, develops with the patient strategies for maintaining change changed behavior |  |  |  |  |
| 16. Comforts the patient, when relapse occurs, in an accepting atmosphere, trying to empower him/her |  |  |  |  |

NA: not applicable, 0: Not at all or insufficiently; 1: Acceptably or enough; 2: To a great extent

Patient’s response:

0. Confrontational 1. Neutral 2. Collaborative
